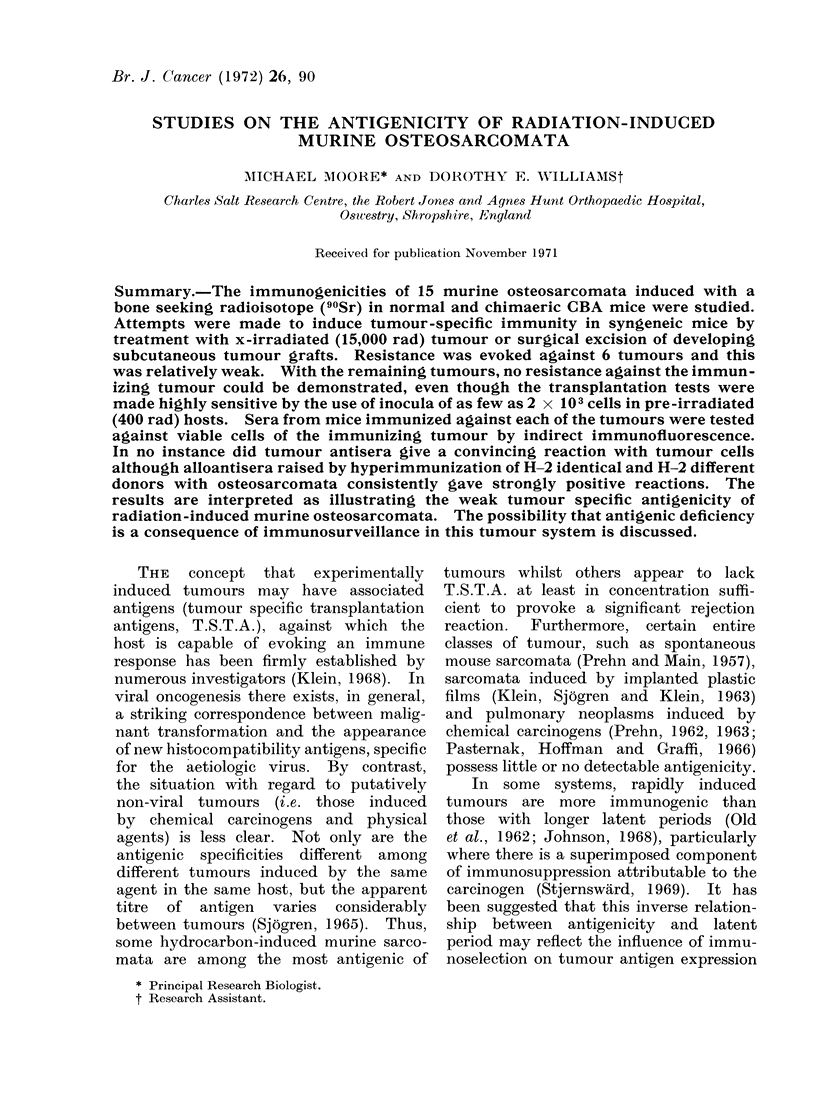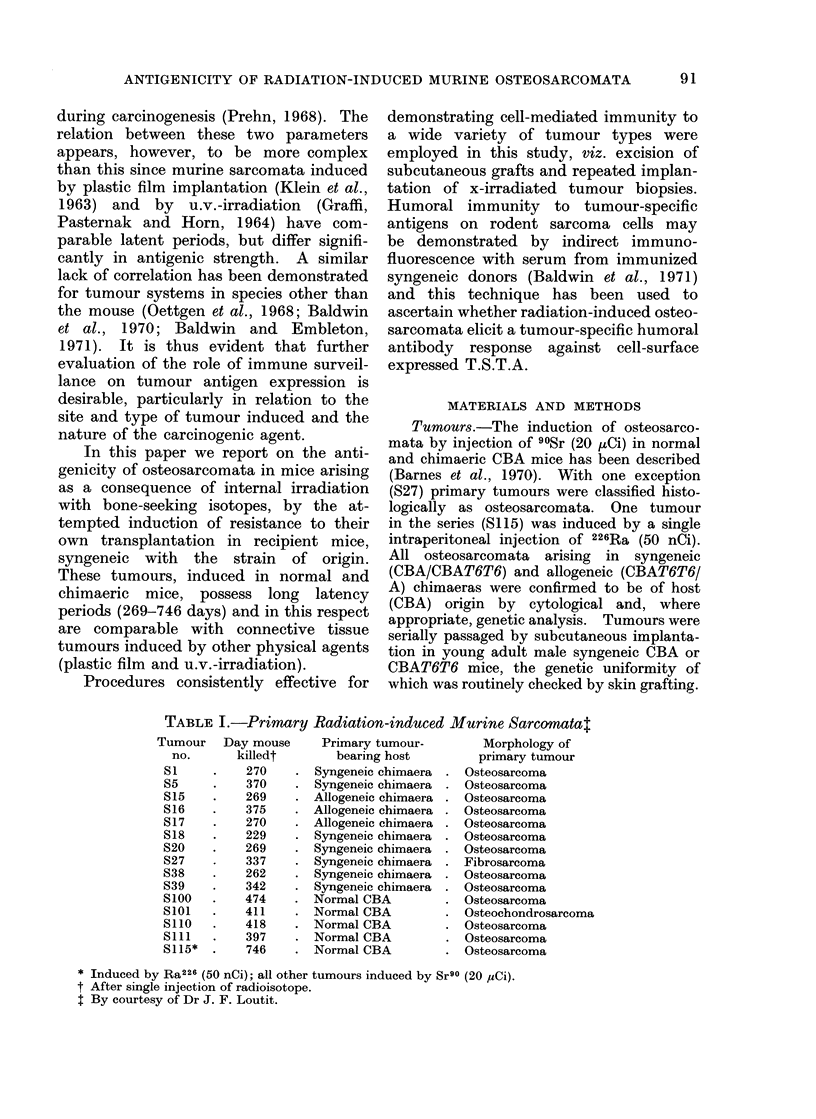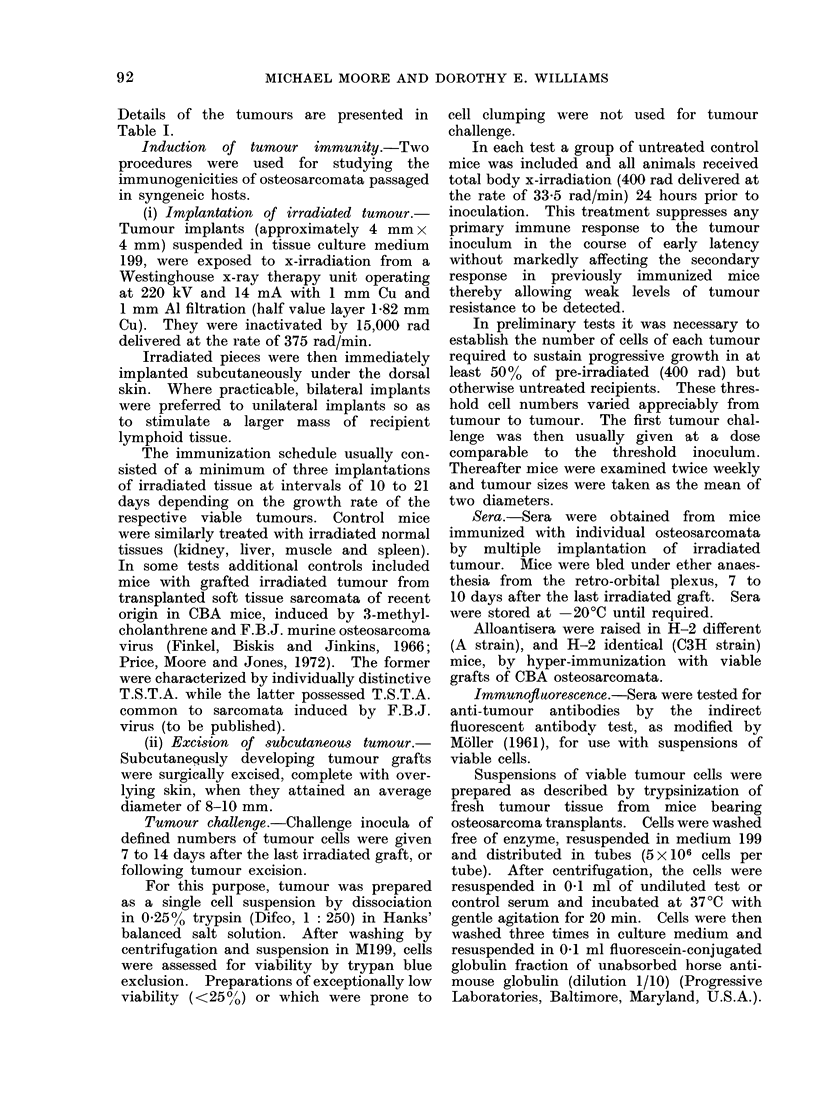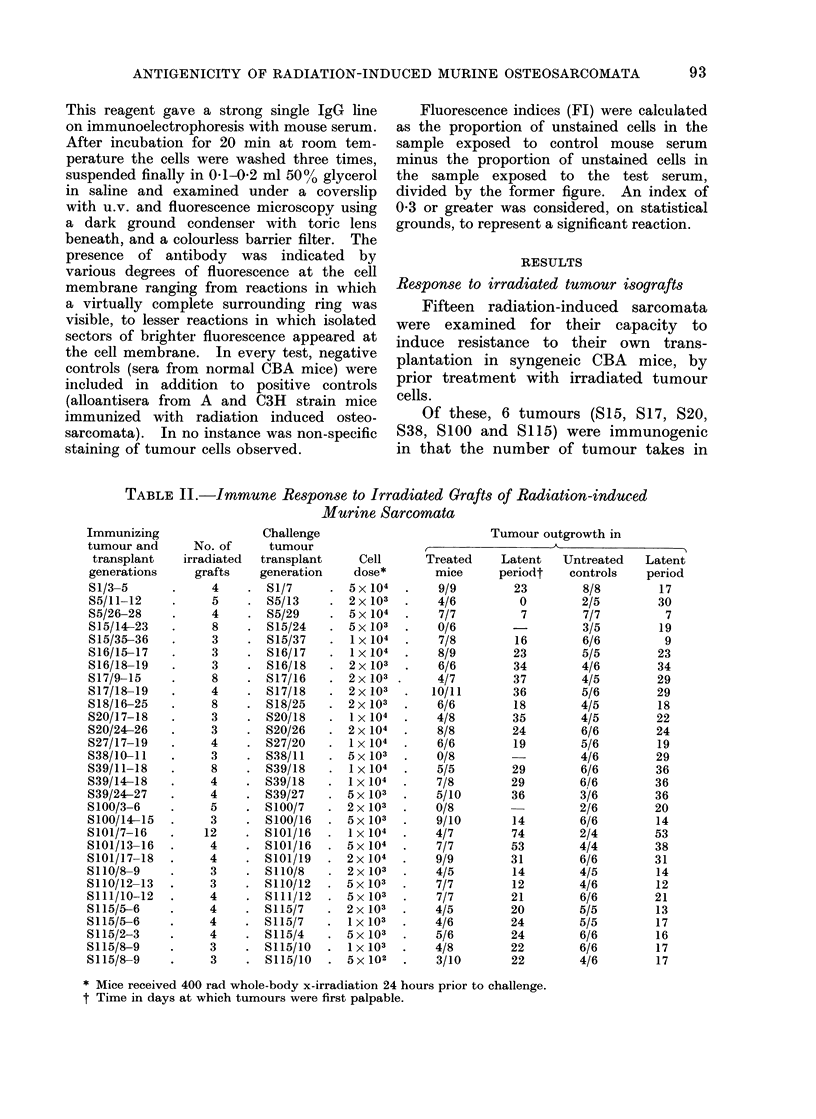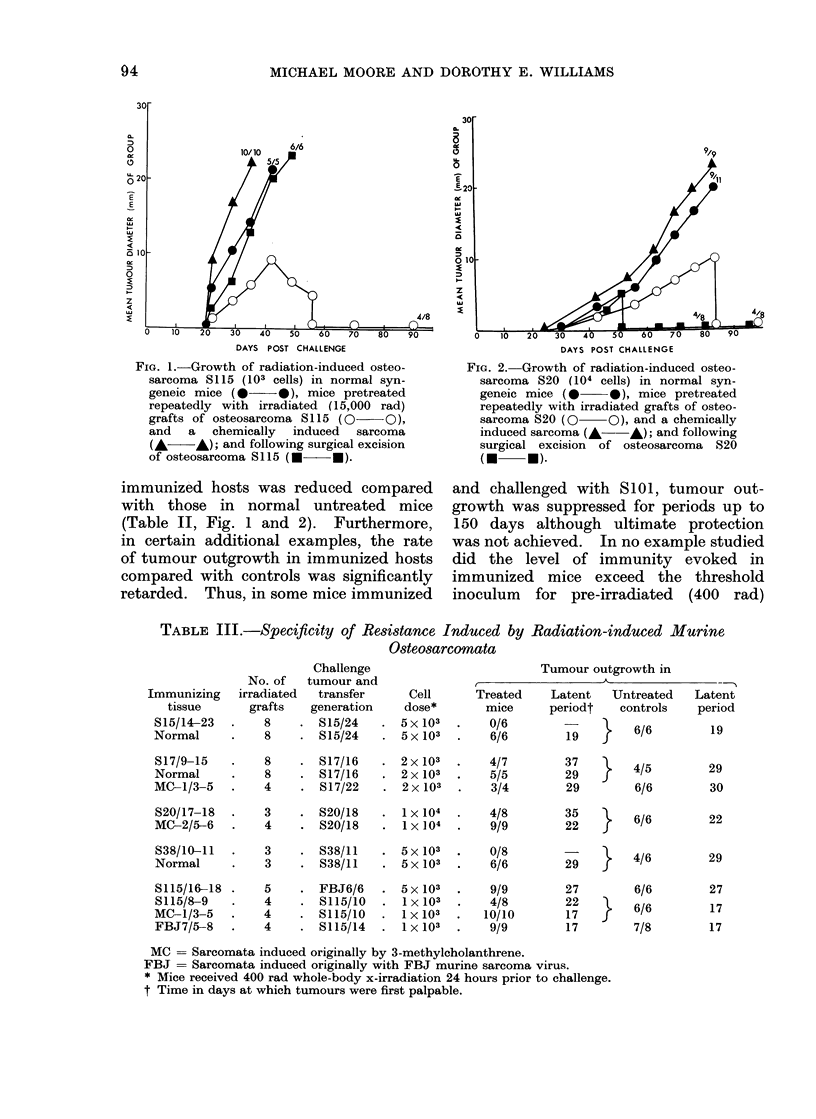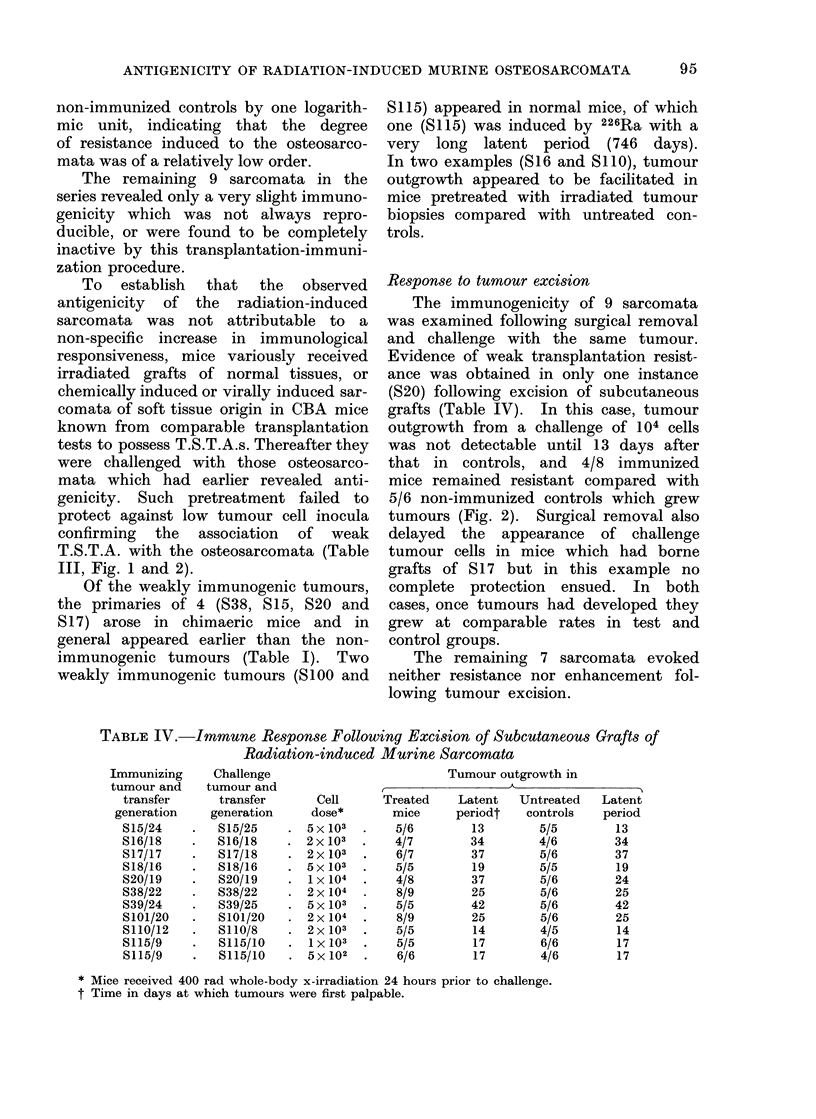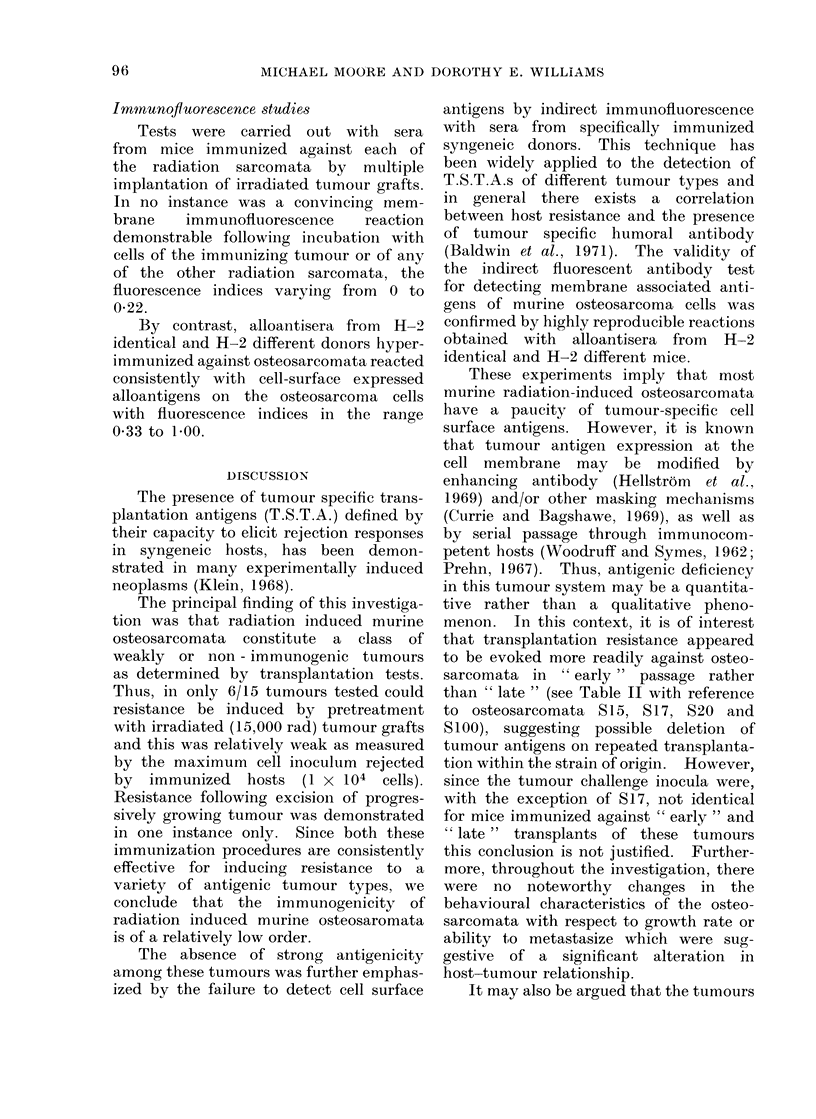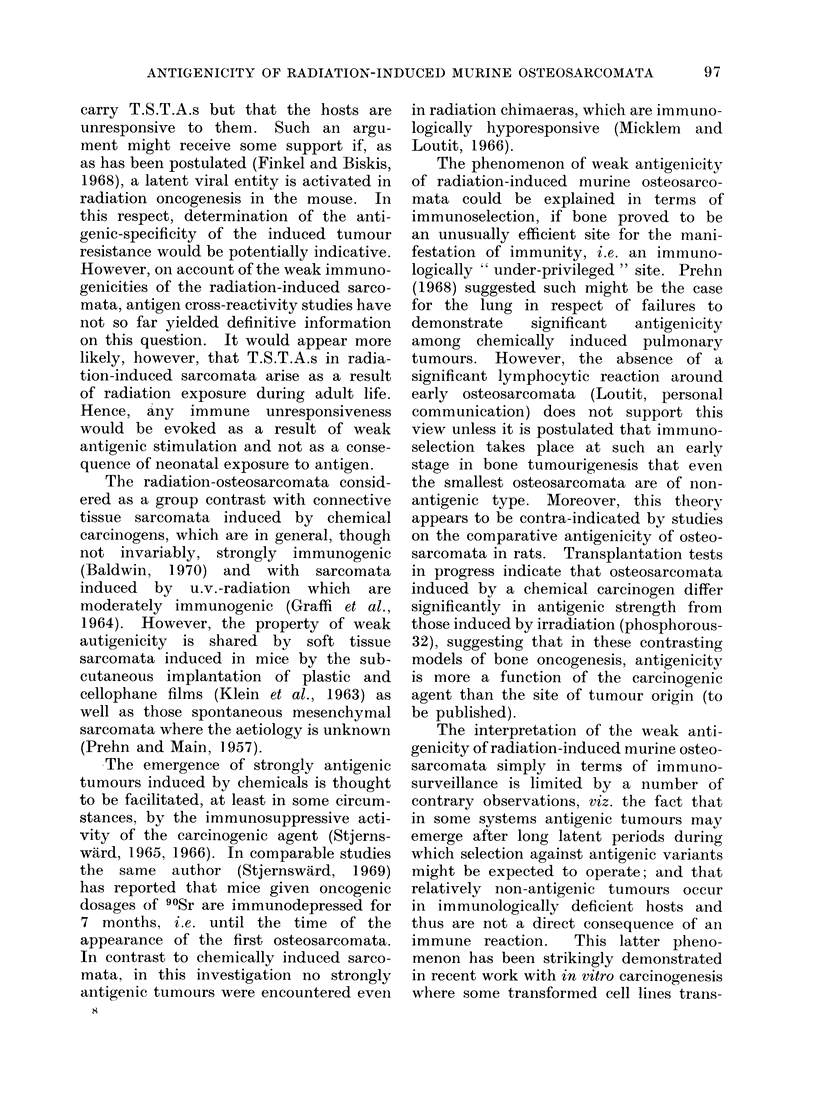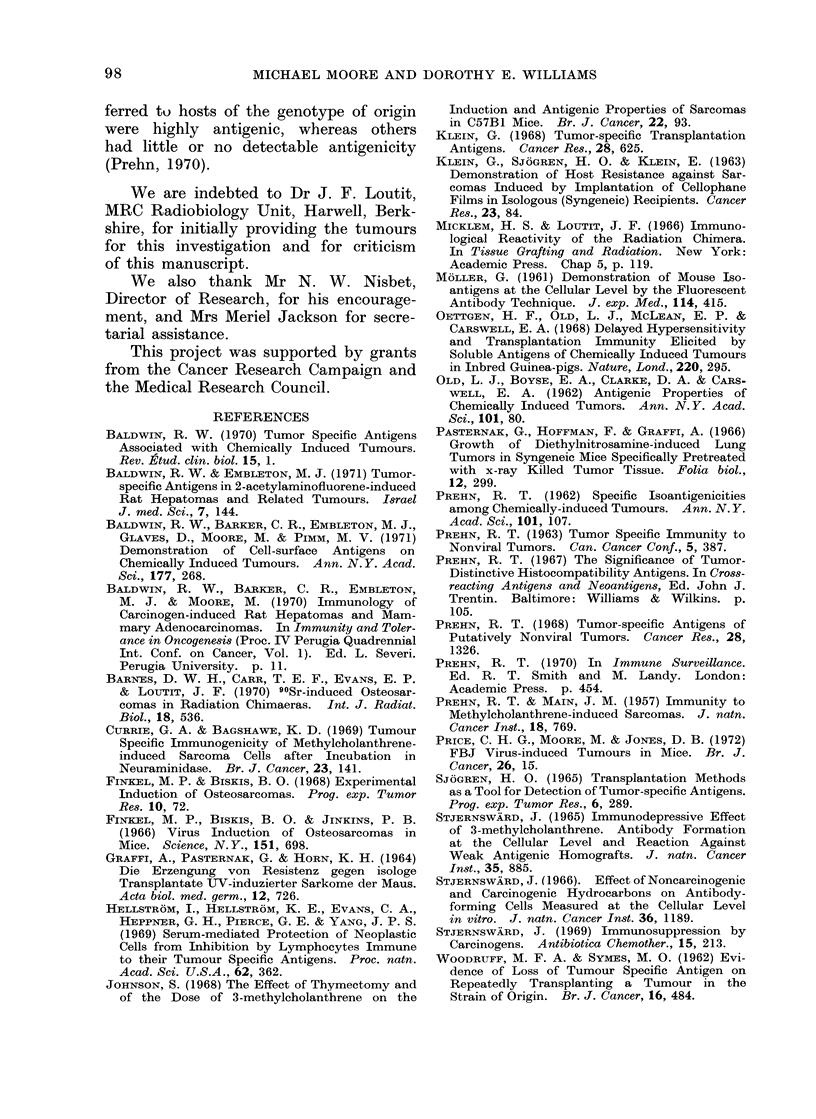# Studies on the Antigenicity of Radiation-induced Murine Osteosarcomata

**DOI:** 10.1038/bjc.1972.14

**Published:** 1972-04

**Authors:** Michael Moore, Dorothy E. Williams

## Abstract

The immunogenicities of 15 murine osteosarcomata induced with a bone seeking radioisotope (^90^Sr) in normal and chimaeric CBA mice were studied. Attempts were made to induce tumour-specific immunity in syngeneic mice by treatment with x-irradiated (15,000 rad) tumour or surgical excision of developing subcutaneous tumour grafts. Resistance was evoked against 6 tumours and this was relatively weak. With the remaining tumours, no resistance against the immunizing tumour could be demonstrated, even though the transplantation tests were made highly sensitive by the use of inocula of as few as 2 × 10^3^ cells in pre-irradiated (400 rad) hosts. Sera from mice immunized against each of the tumours were tested against viable cells of the immunizing tumour by indirect immunofluorescence. In no instance did tumour antisera give a convincing reaction with tumour cells although alloantisera raised by hyperimmunization of H-2 identical and H-2 different donors with osteosarcomata consistently gave strongly positive reactions. The results are interpreted as illustrating the weak tumour specific antigenicity of radiation-induced murine osteosarcomata. The possibility that antigenic deficiency is a consequence of immunosurveillance in this tumour system is discussed.


*Br. J. Cancer* (1972) **26**, 90

### STUDIES ON THE ANTIGENICITY OF RADIATION-INDUCED MURINE OSTEOSARCOMATA

MICHAEL MOORE* AND DOROTHY E. WILLIAMS†

*Charles Salt Research Centre, the Robert Jones and Agnes Hunt Orthopaedic Hospital, Oswestry, Shropshire, England*

Received for publication November 1971

**Summary.—The immunogenicities of 15 murine osteosarcomata induced with a bone seeking radioisotope ($^{90}$Sr) in normal and chimaeric CBA mice were studied. Attempts were made to induce tumour-specific immunity in syngeneic mice by treatment with x-irradiated (15,000 rad) tumour or surgical excision of developing subcutaneous tumour grafts. Resistance was evoked against 6 tumours and this was relatively weak. With the remaining tumours, no resistance against the immunizing tumour could be demonstrated, even though the transplantation tests were made highly sensitive by the use of inocula of as few as $2 \times 10^3$ cells in pre-irradiated (400 rad) hosts. Sera from mice immunized against each of the tumours were tested against viable cells of the immunizing tumour by indirect immunofluorescence. In no instance did tumour antisera give a convincing reaction with tumour cells although alloantisera raised by hyperimmunization of H–2 identical and H–2 different donors with osteosarcomata consistently gave strongly positive reactions. The results are interpreted as illustrating the weak tumour specific antigenicity of radiation-induced murine osteosarcomata. The possibility that antigenic deficiency is a consequence of immunosurveillance in this tumour system is discussed.**

THE concept that experimentally induced tumours may have associated antigens (tumour specific transplantation antigens, T.S.T.A.), against which the host is capable of evoking an immune response has been firmly established by numerous investigators (Klein, 1968). In viral oncogenesis there exists, in general, a striking correspondence between malignant transformation and the appearance of new histocompatibility antigens, specific for the aetiologic virus. By contrast, the situation with regard to putatively non-viral tumours (*i.e.* those induced by chemical carcinogens and physical agents) is less clear. Not only are the antigenic specificities different among different tumours induced by the same agent in the same host, but the apparent titre of antigen varies considerably between tumours (Sjögren, 1965). Thus, some hydrocarbon-induced murine sarcomata are among the most antigenic of tumours whilst others appear to lack T.S.T.A. at least in concentration sufficient to provoke a significant rejection reaction. Furthermore, certain entire classes of tumour, such as spontaneous mouse sarcomata (Prehn and Main, 1957), sarcomata induced by implanted plastic films (Klein, Sjögren and Klein, 1963) and pulmonary neoplasms induced by chemical carcinogens (Prehn, 1962, 1963; Pasternak, Hoffman and Graffi, 1966) possess little or no detectable antigenicity.

In some systems, rapidly induced tumours are more immunogenic than those with longer latent periods (Old *et al.*, 1962; Johnson, 1968), particularly where there is a superimposed component of immunosuppression attributable to the carcinogen (Stjernswärd, 1969). It has been suggested that this inverse relationship between antigenicity and latent period may reflect the influence of immunoselection on tumour antigen expression

* Principal Research Biologist.
† Research Assistant.

during carcinogenesis (Prehn, 1968). The relation between these two parameters appears, however, to be more complex than this since murine sarcomata induced by plastic film implantation (Klein *et al.*, 1963) and by u.v.-irradiation (Graffi, Pasternak and Horn, 1964) have comparable latent periods, but differ significantly in antigenic strength. A similar lack of correlation has been demonstrated for tumour systems in species other than the mouse (Oettgen *et al.*, 1968; Baldwin *et al.*, 1970; Baldwin and Embleton, 1971). It is thus evident that further evaluation of the role of immune surveillance on tumour antigen expression is desirable, particularly in relation to the site and type of tumour induced and the nature of the carcinogenic agent.

In this paper we report on the antigenicity of osteosarcomata in mice arising as a consequence of internal irradiation with bone-seeking isotopes, by the attempted induction of resistance to their own transplantation in recipient mice, syngeneic with the strain of origin. These tumours, induced in normal and chimaeric mice, possess long latency periods (269–746 days) and in this respect are comparable with connective tissue tumours induced by other physical agents (plastic film and u.v.-irradiation).

Procedures consistently effective for demonstrating cell-mediated immunity to a wide variety of tumour types were employed in this study, *viz.* excision of subcutaneous grafts and repeated implantation of x-irradiated tumour biopsies. Humoral immunity to tumour-specific antigens on rodent sarcoma cells may be demonstrated by indirect immunofluorescence with serum from immunized syngeneic donors (Baldwin *et al.*, 1971) and this technique has been used to ascertain whether radiation-induced osteosarcomata elicit a tumour-specific humoral antibody response against cell-surface expressed T.S.T.A.

### MATERIALS AND METHODS

*Tumours.*—The induction of osteosarcomata by injection of $^{90}Sr$ (20 $\mu$Ci) in normal and chimaeric CBA mice has been described (Barnes *et al.*, 1970). With one exception (S27) primary tumours were classified histologically as osteosarcomata. One tumour in the series (S115) was induced by a single intraperitoneal injection of $^{226}Ra$ (50 nCi). All osteosarcomata arising in syngeneic (CBA/CBA*T6T6*) and allogeneic (CBA*T6T6*/A) chimaeras were confirmed to be of host (CBA) origin by cytological and, where appropriate, genetic analysis. Tumours were serially passaged by subcutaneous implantation in young adult male syngeneic CBA or CBA*T6T6* mice, the genetic uniformity of which was routinely checked by skin grafting.

TABLE I.—*Primary Radiation-induced Murine Sarcomata*‡

| Tumour no. | Day mouse killed† | Primary tumour-bearing host | Morphology of primary tumour |
|---|---|---|---|
| S1 | 270 | Syngeneic chimaera | Osteosarcoma |
| S5 | 370 | Syngeneic chimaera | Osteosarcoma |
| S15 | 269 | Allogeneic chimaera | Osteosarcoma |
| S16 | 375 | Allogeneic chimaera | Osteosarcoma |
| S17 | 270 | Allogeneic chimaera | Osteosarcoma |
| S18 | 229 | Syngeneic chimaera | Osteosarcoma |
| S20 | 269 | Syngeneic chimaera | Osteosarcoma |
| S27 | 337 | Syngeneic chimaera | Fibrosarcoma |
| S38 | 262 | Syngeneic chimaera | Osteosarcoma |
| S39 | 342 | Syngeneic chimaera | Osteosarcoma |
| S100 | 474 | Normal CBA | Osteosarcoma |
| S101 | 411 | Normal CBA | Osteochondrosarcoma |
| S110 | 418 | Normal CBA | Osteosarcoma |
| S111 | 397 | Normal CBA | Osteosarcoma |
| S115* | 746 | Normal CBA | Osteosarcoma |

* Induced by $Ra^{226}$ (50 nCi); all other tumours induced by $Sr^{90}$ (20 $\mu$Ci).
† After single injection of radioisotope.
‡ By courtesy of Dr J. F. Loutit.

Details of the tumours are presented in Table I.

*Induction of tumour immunity.*—Two procedures were used for studying the immunogenicities of osteosarcomata passaged in syngeneic hosts.

(i) *Implantation of irradiated tumour.*—Tumour implants (approximately 4 mm × 4 mm) suspended in tissue culture medium 199, were exposed to x-irradiation from a Westinghouse x-ray therapy unit operating at 220 kV and 14 mA with 1 mm Cu and 1 mm Al filtration (half value layer 1·82 mm Cu). They were inactivated by 15,000 rad delivered at the rate of 375 rad/min.

Irradiated pieces were then immediately implanted subcutaneously under the dorsal skin. Where practicable, bilateral implants were preferred to unilateral implants so as to stimulate a larger mass of recipient lymphoid tissue.

The immunization schedule usually consisted of a minimum of three implantations of irradiated tissue at intervals of 10 to 21 days depending on the growth rate of the respective viable tumours. Control mice were similarly treated with irradiated normal tissues (kidney, liver, muscle and spleen). In some tests additional controls included mice with grafted irradiated tumour from transplanted soft tissue sarcomata of recent origin in CBA mice, induced by 3-methylcholanthrene and F.B.J. murine osteosarcoma virus (Finkel, Biskis and Jinkins, 1966; Price, Moore and Jones, 1972). The former were characterized by individually distinctive T.S.T.A. while the latter possessed T.S.T.A. common to sarcomata induced by F.B.J. virus (to be published).

(ii) *Excision of subcutaneous tumour.*—Subcutaneously developing tumour grafts were surgically excised, complete with overlying skin, when they attained an average diameter of 8–10 mm.

*Tumour challenge.*—Challenge inocula of defined numbers of tumour cells were given 7 to 14 days after the last irradiated graft, or following tumour excision.

For this purpose, tumour was prepared as a single cell suspension by dissociation in 0·25% trypsin (Difco, 1 : 250) in Hanks' balanced salt solution. After washing by centrifugation and suspension in M199, cells were assessed for viability by trypan blue exclusion. Preparations of exceptionally low viability (<25%) or which were prone to cell clumping were not used for tumour challenge.

In each test a group of untreated control mice was included and all animals received total body x-irradiation (400 rad delivered at the rate of 33·5 rad/min) 24 hours prior to inoculation. This treatment suppresses any primary immune response to the tumour inoculum in the course of early latency without markedly affecting the secondary response in previously immunized mice thereby allowing weak levels of tumour resistance to be detected.

In preliminary tests it was necessary to establish the number of cells of each tumour required to sustain progressive growth in at least 50% of pre-irradiated (400 rad) but otherwise untreated recipients. These threshold cell numbers varied appreciably from tumour to tumour. The first tumour challenge was then usually given at a dose comparable to the threshold inoculum. Thereafter mice were examined twice weekly and tumour sizes were taken as the mean of two diameters.

*Sera.*—Sera were obtained from mice immunized with individual osteosarcomata by multiple implantation of irradiated tumour. Mice were bled under ether anaesthesia from the retro-orbital plexus, 7 to 10 days after the last irradiated graft. Sera were stored at −20°C until required.

Alloantisera were raised in H–2 different (A strain), and H–2 identical (C3H strain) mice, by hyper-immunization with viable grafts of CBA osteosarcomata.

*Immunofluorescence.*—Sera were tested for anti-tumour antibodies by the indirect fluorescent antibody test, as modified by Möller (1961), for use with suspensions of viable cells.

Suspensions of viable tumour cells were prepared as described by trypsinization of fresh tumour tissue from mice bearing osteosarcoma transplants. Cells were washed free of enzyme, resuspended in medium 199 and distributed in tubes ($5 \times 10^6$ cells per tube). After centrifugation, the cells were resuspended in 0·1 ml of undiluted test or control serum and incubated at 37°C with gentle agitation for 20 min. Cells were then washed three times in culture medium and resuspended in 0·1 ml fluorescein-conjugated globulin fraction of unabsorbed horse anti-mouse globulin (dilution 1/10) (Progressive Laboratories, Baltimore, Maryland, U.S.A.).

This reagent gave a strong single IgG line on immunoelectrophoresis with mouse serum. After incubation for 20 min at room temperature the cells were washed three times, suspended finally in 0·1–0·2 ml 50% glycerol in saline and examined under a coverslip with u.v. and fluorescence microscopy using a dark ground condenser with toric lens beneath, and a colourless barrier filter. The presence of antibody was indicated by various degrees of fluorescence at the cell membrane ranging from reactions in which a virtually complete surrounding ring was visible, to lesser reactions in which isolated sectors of brighter fluorescence appeared at the cell membrane. In every test, negative controls (sera from normal CBA mice) were included in addition to positive controls (alloantisera from A and C3H strain mice immunized with radiation induced osteosarcomata). In no instance was non-specific staining of tumour cells observed.

Fluorescence indices (FI) were calculated as the proportion of unstained cells in the sample exposed to control mouse serum minus the proportion of unstained cells in the sample exposed to the test serum, divided by the former figure. An index of 0·3 or greater was considered, on statistical grounds, to represent a significant reaction.

### RESULTS

### *Response to irradiated tumour isografts*

Fifteen radiation-induced sarcomata were examined for their capacity to induce resistance to their own transplantation in syngeneic CBA mice, by prior treatment with irradiated tumour cells.

Of these, 6 tumours (S15, S17, S20, S38, S100 and S115) were immunogenic in that the number of tumour takes in

TABLE II.—*Immune Response to Irradiated Grafts of Radiation-induced Murine Sarcomata*

| Immunizing tumour and transplant generations | No. of irradiated grafts | Challenge tumour transplant generation | Cell dose* | Tumour outgrowth in: Treated mice | Latent period† | Untreated controls | Latent period |
|---|---|---|---|---|---|---|---|
| S1/3–5 | 4 | S1/7 | $5\times10^4$ | 9/9 | 23 | 8/8 | 17 |
| S5/11–12 | 5 | S5/13 | $2\times10^3$ | 4/6 | 0 | 2/5 | 30 |
| S5/26–28 | 4 | S5/29 | $5\times10^4$ | 7/7 | 7 | 7/7 | 7 |
| S15/14–23 | 8 | S15/24 | $5\times10^3$ | 0/6 | — | 3/5 | 19 |
| S15/35–36 | 3 | S15/37 | $1\times10^4$ | 7/8 | 16 | 6/6 | 9 |
| S16/15–17 | 3 | S16/17 | $1\times10^4$ | 8/9 | 23 | 5/5 | 23 |
| S16/18–19 | 3 | S16/18 | $2\times10^3$ | 6/6 | 34 | 4/6 | 34 |
| S17/9–15 | 8 | S17/16 | $2\times10^3$ | 4/7 | 37 | 4/5 | 29 |
| S17/18–19 | 4 | S17/18 | $2\times10^3$ | 10/11 | 36 | 5/6 | 29 |
| S18/16–25 | 8 | S18/25 | $2\times10^3$ | 6/6 | 18 | 4/5 | 18 |
| S20/17–18 | 3 | S20/18 | $1\times10^4$ | 4/8 | 35 | 4/5 | 22 |
| S20/24–26 | 3 | S20/26 | $2\times10^4$ | 8/8 | 24 | 6/6 | 24 |
| S27/17–19 | 4 | S27/20 | $1\times10^4$ | 6/6 | 19 | 5/6 | 19 |
| S38/10–11 | 3 | S38/11 | $5\times10^3$ | 0/8 | — | 4/6 | 29 |
| S39/11–18 | 8 | S39/18 | $1\times10^4$ | 5/5 | 29 | 6/6 | 36 |
| S39/14–18 | 4 | S39/18 | $1\times10^4$ | 7/8 | 29 | 6/6 | 36 |
| S39/24–27 | 4 | S39/27 | $5\times10^3$ | 5/10 | 36 | 3/6 | 36 |
| S100/3–6 | 5 | S100/7 | $2\times10^3$ | 0/8 | — | 2/6 | 20 |
| S100/14–15 | 3 | S100/16 | $5\times10^3$ | 9/10 | 14 | 6/6 | 14 |
| S101/7–16 | 12 | S101/16 | $1\times10^4$ | 4/7 | 74 | 2/4 | 53 |
| S101/13–16 | 4 | S101/16 | $5\times10^4$ | 7/7 | 53 | 4/4 | 38 |
| S101/17–18 | 4 | S101/19 | $2\times10^4$ | 9/9 | 31 | 6/6 | 31 |
| S110/8–9 | 3 | S110/8 | $2\times10^3$ | 4/5 | 14 | 4/5 | 14 |
| S110/12–13 | 3 | S110/12 | $5\times10^3$ | 7/7 | 12 | 4/6 | 12 |
| S111/10–12 | 4 | S111/12 | $5\times10^3$ | 7/7 | 21 | 6/6 | 21 |
| S115/5–6 | 4 | S115/7 | $2\times10^3$ | 4/5 | 20 | 5/5 | 13 |
| S115/5–6 | 4 | S115/7 | $1\times10^3$ | 4/6 | 24 | 5/5 | 17 |
| S115/2–3 | 4 | S115/4 | $5\times10^3$ | 5/6 | 24 | 6/6 | 16 |
| S115/8–9 | 3 | S115/10 | $1\times10^3$ | 4/8 | 22 | 6/6 | 17 |
| S115/8–9 | 3 | S115/10 | $5\times10^2$ | 3/10 | 22 | 4/6 | 17 |

* Mice received 400 rad whole-body x-irradiation 24 hours prior to challenge.
† Time in days at which tumours were first palpable.

FIG. 1.—Growth of radiation-induced osteosarcoma S115 ($10^3$ cells) in normal syngeneic mice (●——●), mice pretreated repeatedly with irradiated (15,000 rad) grafts of osteosarcoma S115 (○——○), and a chemically induced sarcoma (▲——▲); and following surgical excision of osteosarcoma S115 (■——■).

FIG. 2.—Growth of radiation-induced osteosarcoma S20 ($10^4$ cells) in normal syngeneic mice (●——●), mice pretreated repeatedly with irradiated grafts of osteosarcoma S20 (○——○), and a chemically induced sarcoma (▲——▲); and following surgical excision of osteosarcoma S20 (■——■).

immunized hosts was reduced compared with those in normal untreated mice (Table II, Fig. 1 and 2). Furthermore, in certain additional examples, the rate of tumour outgrowth in immunized hosts compared with controls was significantly retarded. Thus, in some mice immunized and challenged with S101, tumour outgrowth was suppressed for periods up to 150 days although ultimate protection was not achieved. In no example studied did the level of immunity evoked in immunized mice exceed the threshold inoculum for pre-irradiated (400 rad)

TABLE III.—*Specificity of Resistance Induced by Radiation-induced Murine Osteosarcomata*

| Immunizing tissue | No. of irradiated grafts | Challenge tumour and transfer generation | Cell dose* | Tumour outgrowth in: Treated mice | Latent period† | Untreated controls | Latent period |
|---|---|---|---|---|---|---|---|
| S15/14–23 | 8 | S15/24 | $5\times10^3$ | 0/6 | — | 6/6 | 19 |
| Normal | 8 | S15/24 | $5\times10^3$ | 6/6 | 19 | | |
| S17/9–15 | 8 | S17/16 | $2\times10^3$ | 4/7 | 37 | 4/5 | 29 |
| Normal | 8 | S17/16 | $2\times10^3$ | 5/5 | 29 | | |
| MC–1/3–5 | 4 | S17/22 | $2\times10^3$ | 3/4 | 29 | 6/6 | 30 |
| S20/17–18 | 3 | S20/18 | $1\times10^4$ | 4/8 | 35 | 6/6 | 22 |
| MC–2/5–6 | 4 | S20/18 | $1\times10^4$ | 9/9 | 22 | | |
| S38/10–11 | 3 | S38/11 | $5\times10^3$ | 0/8 | — | 4/6 | 29 |
| Normal | 3 | S38/11 | $5\times10^3$ | 6/6 | 29 | | |
| S115/16–18 | 5 | FBJ6/6 | $5\times10^3$ | 9/9 | 27 | 6/6 | 27 |
| S115/8–9 | 4 | S115/10 | $1\times10^3$ | 4/8 | 22 | 6/6 | 17 |
| MC–1/3–5 | 4 | S115/10 | $1\times10^3$ | 10/10 | 17 | | |
| FBJ7/5–8 | 4 | S115/14 | $1\times10^3$ | 9/9 | 17 | 7/8 | 17 |

MC = Sarcomata induced originally by 3-methylcholanthrene.
FBJ = Sarcomata induced originally with FBJ murine sarcoma virus.
* Mice received 400 rad whole-body x-irradiation 24 hours prior to challenge.
† Time in days at which tumours were first palpable.

non-immunized controls by one logarithmic unit, indicating that the degree of resistance induced to the osteosarcomata was of a relatively low order.

The remaining 9 sarcomata in the series revealed only a very slight immunogenicity which was not always reproducible, or were found to be completely inactive by this transplantation-immunization procedure.

To establish that the observed antigenicity of the radiation-induced sarcomata was not attributable to a non-specific increase in immunological responsiveness, mice variously received irradiated grafts of normal tissues, or chemically induced or virally induced sarcomata of soft tissue origin in CBA mice known from comparable transplantation tests to possess T.S.T.A.s. Thereafter they were challenged with those osteosarcomata which had earlier revealed antigenicity. Such pretreatment failed to protect against low tumour cell inocula confirming the association of weak T.S.T.A. with the osteosarcomata (Table III, Fig. 1 and 2).

Of the weakly immunogenic tumours, the primaries of 4 (S38, S15, S20 and S17) arose in chimaeric mice and in general appeared earlier than the non-immunogenic tumours (Table I). Two weakly immunogenic tumours (S100 and S115) appeared in normal mice, of which one (S115) was induced by $^{226}Ra$ with a very long latent period (746 days). In two examples (S16 and S110), tumour outgrowth appeared to be facilitated in mice pretreated with irradiated tumour biopsies compared with untreated controls.

*Response to tumour excision*

The immunogenicity of 9 sarcomata was examined following surgical removal and challenge with the same tumour. Evidence of weak transplantation resistance was obtained in only one instance (S20) following excision of subcutaneous grafts (Table IV). In this case, tumour outgrowth from a challenge of $10^4$ cells was not detectable until 13 days after that in controls, and 4/8 immunized mice remained resistant compared with 5/6 non-immunized controls which grew tumours (Fig. 2). Surgical removal also delayed the appearance of challenge tumour cells in mice which had borne grafts of S17 but in this example no complete protection ensued. In both cases, once tumours had developed they grew at comparable rates in test and control groups.

The remaining 7 sarcomata evoked neither resistance nor enhancement following tumour excision.

TABLE IV.—*Immune Response Following Excision of Subcutaneous Grafts of Radiation-induced Murine Sarcomata*

| Immunizing tumour and transfer generation | Challenge tumour and transfer generation | Cell dose* | Tumour outgrowth in: Treated mice | Latent period† | Untreated controls | Latent period |
|---|---|---|---|---|---|---|
| S15/24 | S15/25 | $5\times10^3$ | 5/6 | 13 | 5/5 | 13 |
| S16/18 | S16/18 | $2\times10^3$ | 4/7 | 34 | 4/6 | 34 |
| S17/17 | S17/18 | $2\times10^3$ | 6/7 | 37 | 5/6 | 37 |
| S18/16 | S18/16 | $5\times10^3$ | 5/5 | 19 | 5/5 | 19 |
| S20/19 | S20/19 | $1\times10^4$ | 4/8 | 37 | 5/6 | 24 |
| S38/22 | S38/22 | $2\times10^4$ | 8/9 | 25 | 5/6 | 25 |
| S39/24 | S39/25 | $5\times10^3$ | 5/5 | 42 | 5/6 | 42 |
| S101/20 | S101/20 | $2\times10^4$ | 8/9 | 25 | 5/6 | 25 |
| S110/12 | S110/8 | $2\times10^3$ | 5/5 | 14 | 4/5 | 14 |
| S115/9 | S115/10 | $1\times10^3$ | 5/5 | 17 | 6/6 | 17 |
| S115/9 | S115/10 | $5\times10^2$ | 6/6 | 17 | 4/6 | 17 |

* Mice received 400 rad whole-body x-irradiation 24 hours prior to challenge.
† Time in days at which tumours were first palpable.

*Immunofluorescence studies*

Tests were carried out with sera from mice immunized against each of the radiation sarcomata by multiple implantation of irradiated tumour grafts. In no instance was a convincing membrane immunofluorescence reaction demonstrable following incubation with cells of the immunizing tumour or of any of the other radiation sarcomata, the fluorescence indices varying from 0 to 0·22.

By contrast, alloantisera from H–2 identical and H–2 different donors hyperimmunized against osteosarcomata reacted consistently with cell-surface expressed alloantigens on the osteosarcoma cells with fluorescence indices in the range 0·33 to 1·00.

### DISCUSSION

The presence of tumour specific transplantation antigens (T.S.T.A.) defined by their capacity to elicit rejection responses in syngeneic hosts, has been demonstrated in many experimentally induced neoplasms (Klein, 1968).

The principal finding of this investigation was that radiation induced murine osteosarcomata constitute a class of weakly or non - immunogenic tumours as determined by transplantation tests. Thus, in only 6/15 tumours tested could resistance be induced by pretreatment with irradiated (15,000 rad) tumour grafts and this was relatively weak as measured by the maximum cell inoculum rejected by immunized hosts ($1 \times 10^4$ cells). Resistance following excision of progressively growing tumour was demonstrated in one instance only. Since both these immunization procedures are consistently effective for inducing resistance to a variety of antigenic tumour types, we conclude that the immunogenicity of radiation induced murine osteosaromata is of a relatively low order.

The absence of strong antigenicity among these tumours was further emphasized by the failure to detect cell surface antigens by indirect immunofluorescence with sera from specifically immunized syngeneic donors. This technique has been widely applied to the detection of T.S.T.A.s of different tumour types and in general there exists a correlation between host resistance and the presence of tumour specific humoral antibody (Baldwin *et al.*, 1971). The validity of the indirect fluorescent antibody test for detecting membrane associated antigens of murine osteosarcoma cells was confirmed by highly reproducible reactions obtained with alloantisera from H–2 identical and H–2 different mice.

These experiments imply that most murine radiation-induced osteosarcomata have a paucity of tumour-specific cell surface antigens. However, it is known that tumour antigen expression at the cell membrane may be modified by enhancing antibody (Hellström *et al.*, 1969) and/or other masking mechanisms (Currie and Bagshawe, 1969), as well as by serial passage through immunocompetent hosts (Woodruff and Symes, 1962; Prehn, 1967). Thus, antigenic deficiency in this tumour system may be a quantitative rather than a qualitative phenomenon. In this context, it is of interest that transplantation resistance appeared to be evoked more readily against osteosarcomata in " early " passage rather than " late " (see Table II with reference to osteosarcomata S15, S17, S20 and S100), suggesting possible deletion of tumour antigens on repeated transplantation within the strain of origin. However, since the tumour challenge inocula were, with the exception of S17, not identical for mice immunized against " early " and " late " transplants of these tumours this conclusion is not justified. Furthermore, throughout the investigation, there were no noteworthy changes in the behavioural characteristics of the osteosarcomata with respect to growth rate or ability to metastasize which were suggestive of a significant alteration in host–tumour relationship.

It may also be argued that the tumours carry T.S.T.A.s but that the hosts are unresponsive to them. Such an argument might receive some support if, as as has been postulated (Finkel and Biskis, 1968), a latent viral entity is activated in radiation oncogenesis in the mouse. In this respect, determination of the antigenic-specificity of the induced tumour resistance would be potentially indicative. However, on account of the weak immunogenicities of the radiation-induced sarcomata, antigen cross-reactivity studies have not so far yielded definitive information on this question. It would appear more likely, however, that T.S.T.A.s in radiation-induced sarcomata arise as a result of radiation exposure during adult life. Hence, any immune unresponsiveness would be evoked as a result of weak antigenic stimulation and not as a consequence of neonatal exposure to antigen.

The radiation-osteosarcomata considered as a group contrast with connective tissue sarcomata induced by chemical carcinogens, which are in general, though not invariably, strongly immunogenic (Baldwin, 1970) and with sarcomata induced by u.v.-radiation which are moderately immunogenic (Graffi *et al.*, 1964). However, the property of weak autigenicity is shared by soft tissue sarcomata induced in mice by the subcutaneous implantation of plastic and cellophane films (Klein *et al.*, 1963) as well as those spontaneous mesenchymal sarcomata where the aetiology is unknown (Prehn and Main, 1957).

The emergence of strongly antigenic tumours induced by chemicals is thought to be facilitated, at least in some circumstances, by the immunosuppressive activity of the carcinogenic agent (Stjernswärd, 1965, 1966). In comparable studies the same author (Stjernswärd, 1969) has reported that mice given oncogenic dosages of $^{90}$Sr are immunodepressed for 7 months, *i.e.* until the time of the appearance of the first osteosarcomata. In contrast to chemically induced sarcomata, in this investigation no strongly antigenic tumours were encountered even in radiation chimaeras, which are immunologically hyporesponsive (Micklem and Loutit, 1966).

The phenomenon of weak antigenicity of radiation-induced murine osteosarcomata could be explained in terms of immunoselection, if bone proved to be an unusually efficient site for the manifestation of immunity, *i.e.* an immunologically " under-privileged " site. Prehn (1968) suggested such might be the case for the lung in respect of failures to demonstrate significant antigenicity among chemically induced pulmonary tumours. However, the absence of a significant lymphocytic reaction around early osteosarcomata (Loutit, personal communication) does not support this view unless it is postulated that immunoselection takes place at such an early stage in bone tumourigenesis that even the smallest osteosarcomata are of non-antigenic type. Moreover, this theory appears to be contra-indicated by studies on the comparative antigenicity of osteosarcomata in rats. Transplantation tests in progress indicate that osteosarcomata induced by a chemical carcinogen differ significantly in antigenic strength from those induced by irradiation (phosphorous-32), suggesting that in these contrasting models of bone oncogenesis, antigenicity is more a function of the carcinogenic agent than the site of tumour origin (to be published).

The interpretation of the weak antigenicity of radiation-induced murine osteosarcomata simply in terms of immunosurveillance is limited by a number of contrary observations, *viz.* the fact that in some systems antigenic tumours may emerge after long latent periods during which selection against antigenic variants might be expected to operate; and that relatively non-antigenic tumours occur in immunologically deficient hosts and thus are not a direct consequence of an immune reaction. This latter phenomenon has been strikingly demonstrated in recent work with *in vitro* carcinogenesis where some transformed cell lines transferred to hosts of the genotype of origin were highly antigenic, whereas others had little or no detectable antigenicity (Prehn, 1970).

We are indebted to Dr J. F. Loutit, MRC Radiobiology Unit, Harwell, Berkshire, for initially providing the tumours for this investigation and for criticism of this manuscript.

We also thank Mr N. W. Nisbet, Director of Research, for his encouragement, and Mrs Meriel Jackson for secretarial assistance.

This project was supported by grants from the Cancer Research Campaign and the Medical Research Council.

### REFERENCES

BALDWIN, R. W. (1970) Tumor Specific Antigens Associated with Chemically Induced Tumours. *Rev. Étud. clin. biol.* **15**, 1.

BALDWIN, R. W. & EMBLETON, M. J. (1971) Tumor-specific Antigens in 2-acetylaminofluorene-induced Rat Hepatomas and Related Tumours. *Israel J. med. Sci.*, **7**, 144.

BALDWIN, R. W., BARKER, C. R., EMBLETON, M. J., GLAVES, D., MOORE, M. & PIMM, M. V. (1971) Demonstration of Cell-surface Antigens on Chemically Induced Tumours. *Ann. N.Y. Acad. Sci.*, **177**, 268.

BALDWIN, R. W., BARKER, C. R., EMBLETON, M. J. & MOORE, M. (1970) Immunology of Carcinogen-induced Rat Hepatomas and Mammary Adenocarcinomas. In *Immunity and Tolerance in Oncogenesis* (Proc. IV Perugia Quadrennial Int. Conf. on Cancer, Vol. 1). Ed. L. Severi. Perugia University. p. 11.

BARNES, D. W. H., CARR, T. E. F., EVANS, E. P. & LOUTIT, J. F. (1970) $^{90}$Sr-induced Osteosarcomas in Radiation Chimaeras. *Int. J. Radiat. Biol.*, **18**, 536.

CURRIE, G. A. & BAGSHAWE, K. D. (1969) Tumour Specific Immunogenicity of Methylcholanthrene-induced Sarcoma Cells after Incubation in Neuraminidase. *Br. J. Cancer*, **23**, 141.

FINKEL, M. P. & BISKIS, B. O. (1968) Experimental Induction of Osteosarcomas. *Prog. exp. Tumor Res.* **10**, 72.

FINKEL, M. P., BISKIS, B. O. & JINKINS, P. B. (1966) Virus Induction of Osteosarcomas in Mice. *Science, N.Y.*, **151**, 698.

GRAFFI, A., PASTERNAK, G. & HORN, K. H. (1964) Die Erzengung von Resistenz gegen isologe Transplantate UV-induzierter Sarkome der Maus. *Acta biol. med. germ.*, **12**, 726.

HELLSTRÖM, I., HELLSTRÖM, K. E., EVANS, C. A., HEPPNER, G. H., PIERCE, G. E. & YANG, J. P. S. (1969) Serum-mediated Protection of Neoplastic Cells from Inhibition by Lymphocytes Immune to their Tumour Specific Antigens. *Proc. natn. Acad. Sci. U.S.A.*, **62**, 362.

JOHNSON, S. (1968) The Effect of Thymectomy and of the Dose of 3-methylcholanthrene on the Induction and Antigenic Properties of Sarcomas in C57Bl Mice. *Br. J. Cancer*, **22**, 93.

KLEIN, G. (1968) Tumor-specific Transplantation Antigens. *Cancer Res.*, **28**, 625.

KLEIN, G., SJÖGREN, H. O. & KLEIN, E. (1963) Demonstration of Host Resistance against Sarcomas Induced by Implantation of Cellophane Films in Isologous (Syngeneic) Recipients. *Cancer Res.*, **23**, 84.

MICKLEM, H. S. & LOUTIT, J. F. (1966) Immunological Reactivity of the Radiation Chimera. In *Tissue Grafting and Radiation*. New York: Academic Press. Chap 5, p. 119.

MÖLLER, G. (1961) Demonstration of Mouse Isoantigens at the Cellular Level by the Fluorescent Antibody Technique. *J. exp. Med.*, **114**, 415.

OETTGEN, H. F., OLD, L. J., MCLEAN, E. P. & CARSWELL, E. A. (1968) Delayed Hypersensitivity and Transplantation Immunity Elicited by Soluble Antigens of Chemically Induced Tumours in Inbred Guinea-pigs. *Nature, Lond.*, **220**, 295.

OLD, L. J., BOYSE, E. A., CLARKE, D. A. & CARSWELL, E. A. (1962) Antigenic Properties of Chemically Induced Tumors. *Ann. N.Y. Acad. Sci.*, **101**, 80.

PASTERNAK, G., HOFFMAN, F. & GRAFFI, A. (1966) Growth of Diethylnitrosamine-induced Lung Tumors in Syngeneic Mice Specifically Pretreated with x-ray Killed Tumor Tissue. *Folia biol.*, **12**, 299.

PREHN, R. T. (1962) Specific Isoantigenicities among Chemically-induced Tumours. *Ann. N.Y. Acad. Sci.*, **101**, 107.

PREHN, R. T. (1963) Tumor Specific Immunity to Nonviral Tumors. *Can. Cancer Conf.*, **5**, 387.

PREHN, R. T. (1967) The Significance of Tumor-Distinctive Histocompatibility Antigens. In *Cross-reacting Antigens and Neoantigens*, Ed. John J. Trentin. Baltimore: Williams & Wilkins. p. 105.

PREHN, R. T. (1968) Tumor-specific Antigens of Putatively Nonviral Tumors. *Cancer Res.*, **28**, 1326.

PREHN, R. T. (1970) In *Immune Surveillance*. Ed. R. T. Smith and M. Landy. London: Academic Press. p. 454.

PREHN, R. T. & MAIN, J. M. (1957) Immunity to Methylcholanthrene-induced Sarcomas. *J. natn. Cancer Inst.*, **18**, 769.

PRICE, C. H. G., MOORE, M. & JONES, D. B. (1972) FBJ Virus-induced Tumours in Mice. *Br. J. Cancer*, **26**, 15.

SJÖGREN, H. O. (1965) Transplantation Methods as a Tool for Detection of Tumor-specific Antigens. *Prog. exp. Tumor Res.*, **6**, 289.

STJERNSWÄRD, J. (1965) Immunodepressive Effect of 3-methylcholanthrene. Antibody Formation at the Cellular Level and Reaction Against Weak Antigenic Homografts. *J. natn. Cancer Inst.*, **35**, 885.

STJERNSWÄRD, J. (1966). Effect of Noncarcinogenic and Carcinogenic Hydrocarbons on Antibody-forming Cells Measured at the Cellular Level *in vitro*. *J. natn. Cancer Inst.* **36**, 1189.

STJERNSWÄRD, J. (1969) Immunosuppression by Carcinogens. *Antibiotica Chemother.*, **15**, 213.

WOODRUFF, M. F. A. & SYMES, M. O. (1962) Evidence of Loss of Tumour Specific Antigen on Repeatedly Transplanting a Tumour in the Strain of Origin. *Br. J. Cancer*, **16**, 484.